# RepSAU-Net: Semantic Segmentation of Barcodes in Complex Backgrounds via Fused Self-Attention and Reparameterization Methods

**DOI:** 10.3390/jimaging11110394

**Published:** 2025-11-06

**Authors:** Yanfei Sun, Junyu Wang, Rui Yin

**Affiliations:** 1 National Integrated Circuit Innovation Center, Shanghai 200120, China; yanfeisun@fudan.edu.cn (Y.S.); junyuwang@fudan.edu.cn (J.W.); 2 Key Laboratory of Integrated Chip and Systems, School of Microelectronics, Fudan University, Shanghai 200433, China

**Keywords:** wide field-of-view images, Image segmentation, U-Net, self-attention mechanism

## Abstract

In the digital era, commodity barcodes serve as a bridge between the physical and digital worlds and are widely used in retail checkout systems. To meet the broader application demands for product identification, this paper proposes a method for locating, semantically segmenting barcodes in complex backgrounds, decoding hidden information, and recovering these barcodes in wide field-of-view images. This method integrates self-attention mechanisms and reparameterization techniques to construct a RepSAU-Net model. Specifically, this paper first introduces a barcode image dataset synthesis strategy adapted for deep learning models, constructing the SBS (Screen Stego Barcodes) dataset, which comprises 2000 wide field-of-view background images (Type A) and 400 information-hidden barcode images (Type B), totaling 30,000 images. Based on this, a network architecture (RepSAU-Net) combining a self-attention mechanism and RepVGG reparameterization technology was designed, with a parameter count of 32.88 M. Experimental results demonstrate that this network performs well in barcode segmentation tasks, achieving an inference speed of 4.88 frames/s, a Mean Intersection over Union (MIoU) of 98.36%, and an Accuracy (Acc) of 94.96%. This research effectively enhances global information capture and feature extraction capabilities without significantly increasing computational load, providing technical support for the application of data-embedded barcodes.

## 1. Introduction

Commodity barcodes, as the internationally recognized identifier for products, represent a printable language widely applied in the retail sector, with a global daily scanning volume exceeding 10 billion [1,2]. Through barcode technology, manufacturers, exporters, wholesalers, retailers, and consumers worldwide are organically connected, forming a diverse information network. This provides a reliable data foundation for brands, retailers, and enterprises, ensuring more reliable, complete, and accurate product identification while enhancing service quality [3,4,5,6]. The international standardization organization for commodity barcodes, GS1, has 115 member organizations globally, underscoring the significant importance of innovative research on barcode images [7,8,9].

However, traditional commodity barcodes have limited information capacity, typically containing only enterprise codes and product category codes, which struggle to meet the growing demand for comprehensive product information services [10,11]. Although technologies such as two-dimensional barcodes and color codes can expand data capacity, their widespread adoption faces challenges. These often require updates to retail scanning equipment, resulting in high implementation costs and long update cycles, and they are often incompatible with existing retail checkout systems [12,13]. Therefore, expanding the data capacity of commodity barcodes while maintaining compatibility with existing automatic identification devices holds significant research significance and application value [14,15,16].

In the field of image segmentation, traditional methods primarily rely on basic visual attributes of pixels, such as approaches based on Texton Forests, Support Vector Machines (SVM), and Conditional Random Fields (CRF) [17,18,19]. These methods often utilize domain-specific knowledge to extract features and require post-processing techniques to complete segmentation tasks. When faced with complex segmentation tasks, their performance is limited without human intervention. In recent years, models based on Convolutional Neural Networks (CNNs) have made significant progress in image segmentation [20,21,22,23,24,25]. Fully Convolutional Networks (FCNs) achieve effective fusion of high-level and low-level features through deconvolution layers, upsampling, and skip connection architectures [26,27,28]. Dilated convolutions and the DeepLab series (v1, v2, v3, v3+) expand the receptive field through consecutive convolutional layers while maintaining image resolution [28,29,30,31,32]. To improve recognition accuracy, researchers have adopted various strategies, including feature integration, multi-stage training, acquiring additional training data from other datasets, object proposals, CRF-based post-processing, and pyramid-based feature resampling [33,34,35,36,37].

However, research on barcode image segmentation remains scarce, with most studies focusing on barcode detection and recognition. Notable works include: Duan et al. [38], who proposed a lightweight two-stage framework for multi-type barcode defect detection. In the first stage, real-time localization of one-dimensional and two-dimensional barcodes is performed. In the second stage, a dual-branch network integrating hierarchical attention with ResNet50 and ViT-B/16 performs three-class classification (complete, defective, and non-barcode) on cropped regions of interest (ROI), achieving high performance. Ouyang et al. [39] introduced a method based on the GooLeNet network for barcode and damage detection on industrial packaging surfaces. Capable of capturing and quickly reading barcodes from six directions, they combined the GooLeNet architecture with regularization to design an improved GooLeNet model, facilitating feature extraction and training under interference from packaging surface patterns, significantly improving recognition accuracy. Qi et al. [40] divided barcode regions into barcode and character parts for defect detection. For the barcode part, Support Vector Machines were used to construct features and classify defects. For the character part, a grayscale projection-based quality evaluation method was adopted to output character quality scores. By combining barcode defect classification results with subjective quality judgments, defect detection in barcode regions on non-planar glass bottle surfaces was achieved. Tang et al. [41] proposed a lightweight real-time QR code detection algorithm, EBP-YOLOv5. This work introduced an efficient channel attention mechanism in the backbone network to enhance feature extraction capabilities. A bidirectional feature pyramid network replaced the PANet module to improve the network’s ability to capture features along paths. Additionally, EBP-YOLOv5 reduced model parameters through sparse training for lightweight design, incorporated L1 regularization into the loss function to prune batch normalization layer weights, and applied distillation learning to improve model accuracy. Extensive evaluation on a natural scene QR code dataset demonstrated the superior performance of EBP-YOLOv5. Selvam et al. [42] proposed an OCR-based object recognizer called Batch Normalization-Free Rigid Feature Flow Neural Network (BNFRNN). The performance of the proposed framework was evaluated on the benchmark dataset WebMarket, outperforming state-of-the-art methods with a barcode recognition accuracy score of 92.56%, a recall rate of 85.64%, and an F-score of 88.97%. Pandya et al. [43] conducted large-scale training and fine-tuning on a Kaggle dataset tailored for barcode and QR code detection, optimizing YOLOv8’s overall performance across various scenarios and environments. The evaluation covered YOLOv8 iterations in nano, small, and medium versions, achieving accuracies of 88.95% for the nano model, 97.10% for the small model, and 94.10% for the medium model. Chen et al. [44] addressed issues of missed, false, and duplicate detections in barcode detection algorithms under real-world scenarios by proposing a barcode detection algorithm incorporating an EfficientViT block based on a linear self-attention mechanism in the backbone of the original model to enhance attention to barcode features. In the neck of the model, linear mapping and grouped convolutions were used to improve the C2f module, while the ADown convolution block was adopted to modify downsampling, reducing model parameters and computational costs while improving feature fusion efficiency. Finally, the detection head was reconstructed, and the loss function was modified to enhance training quality and reduce errors in barcode detection.

However, existing barcode research predominantly focuses on object detection and recognition, with very limited application of image segmentation in this domain. Simple object detection methods typically yield only coarse bounding box localizations for barcodes. However, in many practical applications, precise pixel-level masks are indispensable. For instance, when barcodes are damaged, partially occluded, severely deformed, or embedded within complex backgrounds, accurate pixel-level segmentation enables more precise extraction of the barcode region, effectively eliminating background noise, and thereby providing a clean input for subsequent image correction and decoding processes. Furthermore, precise pixel-level segmentation is adept at handling irregular shapes. Barcodes can exhibit non-rectangular forms due to factors such as paper creases, bending, or perspective distortions, and semantic segmentation is uniquely suited to accurately delineate these non-rectangular regions. Moreover, these accurate masks can serve as a crucial guide for decoding algorithms, directing them to focus exclusively on the barcode region, which significantly enhances decoding success rates, particularly in low-quality or blurry images. Ultimately, semantic segmentation inherently provides richer information compared to simple bounding box detections, which is invaluable for subsequent quality control, anomaly detection, and understanding the barcode’s interaction with its surrounding environment.

Moreover, the aforementioned methods rely on clear barcode image datasets acquired under ideal lighting conditions, which significantly differ from the complexity and diversity of real-world application scenarios. Additionally, these methods struggle to achieve robust barcode segmentation in variable environments. Therefore, prior to scanning and extracting information from barcodes with embedded data, there is an urgent need to develop an effective segmentation method capable of adapting to various imaging conditions and accurately identifying small-sized barcodes. To address this challenge and efficiently recover hidden information from data-embedded barcodes in captured wide field-of-view images, this paper proposes a novel network for detection and semantic segmentation of barcodes in complex backgrounds. This network aims to achieve efficient and accurate detection and precise semantic segmentation of barcodes in complex backgrounds. The main contributions of this paper are as follows:

SBS Dataset: Addressing the lack of high-quality annotated datasets and suitable deep learning models for segmentation tasks involving barcodes with hidden information, this paper proposes an efficient synthetic dataset method and constructs the ScreenBar-Synth (SBS) dataset. Furthermore, we collected 50 real-world barcode images to facilitate robust generalization testing of models. The SBS dataset, comprising approximately 30,000 synthetic barcode images with embedded data and these 50 real-world images, thus provides a solid data foundation for research in this field.

RepSAU-Net: This paper designs and implements a barcode segmentation network (RepSAU-Net) based on U-Net, integrating self-attention mechanisms and RepVGG reparameterization techniques to enhance the model’s generalization ability and segmentation performance.

L_HDB_: This paper proposes a Hybrid Dice-BCE Loss (L_HDB_), which leverages the strength of Dice Loss in optimizing global overlap for small target regions (such as barcodes) while compensating for its lack of sensitivity to pixel-level details. It ingeniously combines BCE Loss to provide precise classification guidance at the pixel level, effectively capturing local features.

## 2. Materials and Methods

### 2.1. ScreenBar-Synth (SBS) Dataset

**Figure 1:**
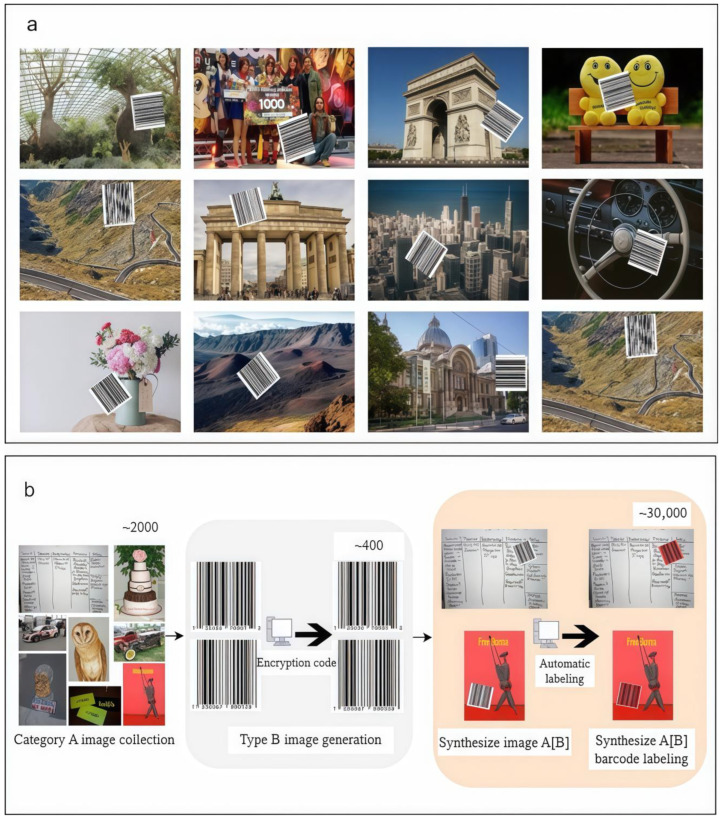
SBS dataset production process (**b**) and sample example (**a**).

Addressing the challenge of scarce high-quality annotated datasets for barcode segmentation tasks involving hidden information, this paper proposes an efficient synthetic dataset generation method and constructs the ScreenBar-Synth (SBS) dataset. An example (a) and the annotation pipeline (b) are illustrated in Figure 1. This dataset comprises approximately 30,000 images simulating commodity barcodes with hidden information displayed on electronic screens, aiming to provide large-scale, high-diversity training resources for deep learning models.

The ScreenBar-Synth (SBS) dataset employs a fully automated pixel-level annotation process, a significant departure from traditional time-consuming and labor-intensive manual annotation paradigms. This study achieves automated and scalable annotation by programmatically superimposing 400 small, information-hidden commodity barcode images (Type B images) onto 2000 relatively larger background images (Type A images). Specifically, this automated synthesis method systematically selects Type B images and overlays them onto pre-defined positions within Type A images. Since the precise positions and pixel-level contours of Type B images are known prior to synthesis, accurate pixel-level labels can be automatically generated without manual intervention, thereby realizing an integrated image synthesis and automatic annotation mechanism. Concurrently, by algorithmically controlling the combination and placement of Type B and Type A images, this automated process can efficiently generate synthetic images with precise annotations in bulk, effectively reducing labor costs and time consumption, demonstrating its capability for large-scale production. Furthermore, to ensure dataset diversity, challenge, and model generalization ability, this study designed and programmatically implemented a series of synthesis rules within this automated framework. These include instance quantity control, ensuring each synthetic image contains at least one Type B instance; randomness simulation for Type B image count, following a Poisson distribution with parameter λ = 2 (upper limit of 10) to mimic the randomness of barcodes in real-world scenarios; and type mixing, automatically blending same and different types of Type B images to increase dataset complexity and realism. These collectively form an integrated strategy for diversity and challenge. The image transformation strategies are as follows:

Broad Scaling Range: S ∈ [0.2, 5], simulating significant variations in barcode size.

Multi-angle Rotation: θ ∈ [−45°, 45°], covering common tilt conditions.

Random Cropping: C ∈ [0.5, 1] (10% probability), simulating partial occlusions.

Illumination and Resolution Adjustment: L ∈ [0.5, 1], increasing robustness to changes in lighting and image quality.

**Figure 2:**
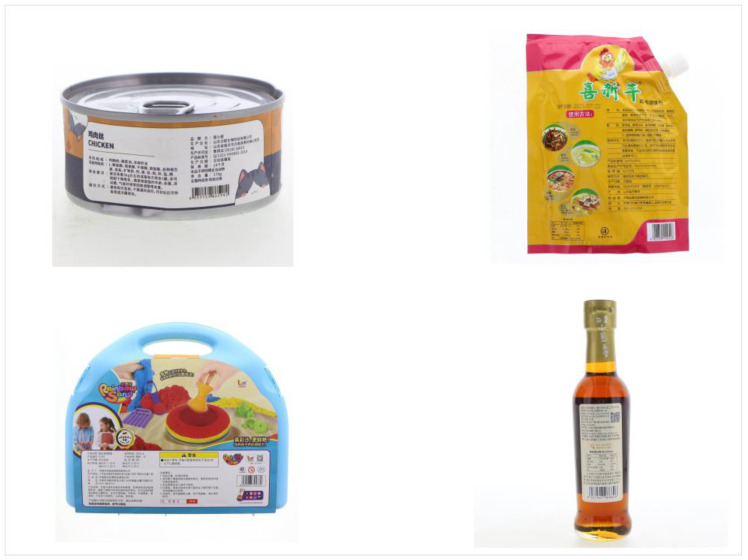
Sample real (Images of real-world products with barcodes).

Furthermore, the SBS Dataset also incorporates 50 real-world barcode images, which were specifically collected to validate the model’s generalization capabilities on authentic barcode segmentation tasks. Illustrative examples are presented in Figure 2.

### 2.2. RepSAU-Net

**Figure 3:**
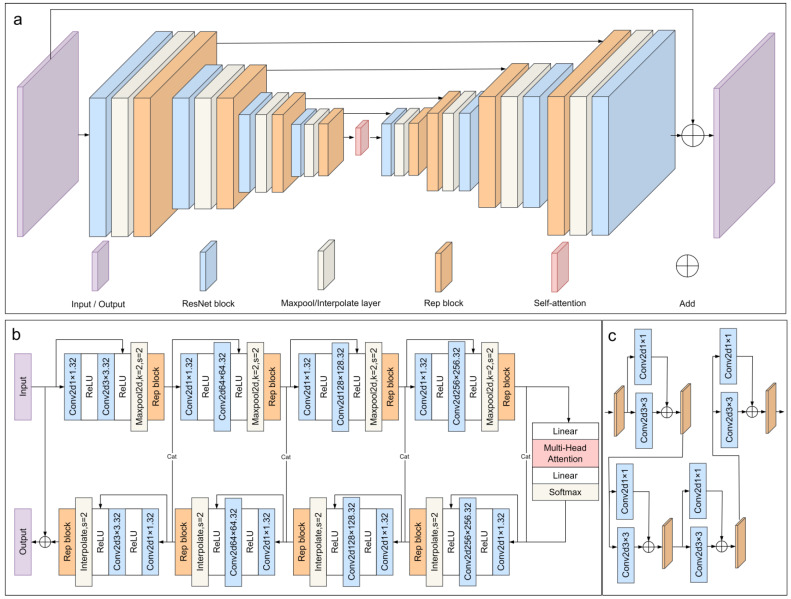
Model structure diagram. (**a**) Overall Architecture; (**b**) Detailed Model Structure; (**c**) RepBlock Structure. Arrows in the diagram indicate data flow.

This paper proposes a novel deep learning network architecture named RepSAU-Net for wide field-of-view barcode image segmentation. Its specific structure is illustrated in Figure 3, where (a) presents the overall architecture, (b) details the model’s structure, and (c) shows the RepBlock structure. This architecture integrates a U-Net-like structure, incorporating self-attention mechanisms and structural reparameterization (RepVGG) for optimized design. At its core is a multi-stage feature extraction and fusion pipeline, engineered to process and analyze input wide field-of-view barcode images containing hidden information, thereby achieving precise barcode segmentation.

The input image first undergoes a 1 × 1 convolutional layer, whose primary role is initial channel adjustment to configure the input channel structure for subsequent deep feature extraction. Subsequently, the image features are fed into RepBlocks, which are composed of RepVGG blocks. These RepBlocks extract key features from the image through feature extraction and non-linear activation. Following processing by each RepBlock, the feature maps undergo a subsampling operation to reduce their spatial dimensions, thereby enhancing computational efficiency and decreasing the number of model parameters. Furthermore, to optimize the feature maps for the network’s next stage, we employ an additional 1 × 1 convolution for channel adjustment. To improve the model’s generalization ability and prevent overfitting, we embed Dropout layers within the network. This layer enhances model robustness by randomly deactivating a fraction of neurons. The channel-adjusted feature maps are then fed into another RepBlock for deeper feature extraction. After continuous subsampling and RepBlock processing, the feature maps are dimensionally adjusted and passed to a Self-attention module. This module aims to capture deep semantic features, leveraging attention mechanisms to strengthen the model’s ability to identify critical information.

Subsequently, the feature maps processed by the Self-attention module undergo further feature extraction and processing through RepBlocks. After channel adjustment via 1 × 1 convolution, the feature maps are upsampled to the previous scale and fused with the corresponding RepBlock output features from that level. This fusion process is iteratively performed across different levels to ensure the richness and preservation of feature map details. Finally, the feature maps, after multiple upsampling and fusion operations, pass through a final 1 × 1 convolution for ultimate channel adjustment, yielding the final prediction results. This multi-stage feature extraction and fusion pipeline is designed to enhance the accuracy of barcode segmentation.

Structural reparameterization technology improves inference speed through multi-branch fusion. The RepVGG network extensively utilizes convolutional layers and Batch Normalization (BN) layers. Its core idea is to fuse these layers during the inference phase, thereby enhancing efficiency. The derivation process is as follows:

The convolution operation can be expressed as:

(1) Y=X*W+B.

where, *X* is the input, *W* is the convolution kernel, *B* is the bias, and * denotes the convolution operation. The BN layer operation can be expressed as:

(2) $$Y=\gamma \frac{X-\mu }{\sqrt{\sigma^{2}+\varepsilon }}+\beta .$$

Among them, *μ* and *σ*^2^ are the mean value and variance, *γ* and *β* are learnable scaling factors and offsets, *ϵ* is a small constant. By substituting the output of the convolutional layer into the BN layer formula, we can obtain:

(3) $$Y=\gamma \frac{(X*W+B)-\mu }{\sqrt{\sigma^{2}+\varepsilon }}+\beta .$$

Simplification gives:

(4) $$Y=X*\frac{\gamma W}{\sqrt{\sigma^{2}+\varepsilon }}+(\beta -\frac{\gamma \mu }{\sqrt{\sigma^{2}+\varepsilon }}+\frac{\gamma B}{\sqrt{\sigma^{2}+\varepsilon }}).$$

Replace with the following formula:

(5) $$W^{\prime }=\frac{\lambda W}{\sqrt{\sigma^{2}+\varepsilon }},$$

(6) $$B^{\prime }=\beta -\frac{\gamma \mu }{\sqrt{\sigma^{2}+\varepsilon }}+\frac{\gamma B}{\sqrt{\sigma^{2}+\varepsilon }},$$

the final result can be simplified as:

(7) $$Y=X*W^{\prime }+B^{\prime }.$$

This implies that a convolutional layer and a Batch Normalization (BN) layer can be equivalently merged into a single convolutional layer with a new convolutional kernel *W*′ and a new bias *B*′. Upon completion of training, the parameters within *W*’ and *B* are determined. Consequently, the final *W*’ and *B* can be computed, enabling the fusion of the convolutional layer and the Batch Normalization layer into a single equivalent convolutional layer. This eliminates the need for discrete Batch Normalization operations during inference, which is precisely how RepVGG achieves its superior inference efficiency.

The reparameterization principle of RepVGG algebraically merges multi-branch structures (e.g., 3 × 3 convolution, 1 × 1 convolution, and identity mapping branches) during the training phase into a single branch for the inference phase. Specifically, a RepVGG block comprises a 3 × 3 convolutional branch, a 1 × 1 convolutional branch, and an identity mapping branch (if input and output channels are identical). During inference, the convolutional kernels and biases of these branches can be algebraically combined to form an equivalent 3 × 3 convolutional kernel and bias, thereby reducing computational cost without sacrificing performance.

The self-attention mechanism primarily captures relationships between different positions within an image by computing attention weights. Its core formula is:

(8) $$Attention(Q,K,V)=soft\max(\frac{QK^{T}}{\sqrt{d_{k}}})V.$$

Here, *Q* represents the query vector, *K* represents the key vector, *V* represents the value vector, and *d_k_* is the dimension of the key vector. $\frac{QK^{T}}{\sqrt{d_{k}}}$ represents the degree of attention of position *i* to position *j*.

In terms of the body, we first transform the image features from the hidden layer in the previous layer into three feature spaces: query (*Q*), key (*K*), and value (*V*). For the input feature map x ∈ R C × H × W, we generate *Q*, *K*, and *V* through a 1 × 1 convolution.

(9) $$f(x)=W_{f}x,g(x)=W_{g}x,h(x)=W_{h}x.$$

where $W_{f}$, $W_{g}$, $W_{h}$ are learnable weight matrices. The attention score is calculated as follows:

(10) $$s_{ij}=f{\left(x_{i}\right)}^{T}g(x_{j}).$$

Then, the attention weights are normalized by the softmax function:

(11) $$\beta_{ij}=\frac{\exp(s_{ij})}{\sum_{k=1}^{N}\exp(s_{ik})}.$$

*N* is the total number of feature positions. The output *o_j_* of the attention layer is given by the following formula:

(12) $$o_{j}=\sum_{i=1}^{N}\beta_{ji}h(x_{i}).$$

Finally, in order to enhance the expressive power of the model, we multiply the output of the attention layer by a learnable proportional parameter *γ* and add it back to the original input feature map

(13) Y=γo+x.

where *γ* is a learnable scalar, initialized to 0. The introduction of a learnable *γ* allows the network to initially rely on cues from the local neighborhood and then gradually learn to assign more weight to non-local evidence.

The self-attention mechanism has demonstrated remarkable success in various advanced architectures for capturing long-range dependencies across wide spatial regions of an image. This capability of non-local modeling, which allows a network to efficiently process connections between spatially distant areas, is particularly relevant to our barcode segmentation task.

### 2.3. Hybrid Dice-BCE Loss

To effectively train the RepSAU-Net model for accurate pixel-level segmentation of barcode regions, we designed a Hybrid Dice-BCE Loss.

In image segmentation tasks, particularly when the target region (e.g., barcode) occupies a small proportion of the image, Dice Loss is considered an effective loss function as it directly measures the overlap between the predicted result and the ground truth label. The formula for Dice Loss is as follows:

(14) $$L_{Dice}=1-\frac{2\sum_{i=1}^{N}p_{i}g_{i}+\varepsilon }{\sum_{i=1}^{N}{p_{i}}^{2}+\sum_{i=1}^{N}{g_{i}}^{2}+\varepsilon }.$$

where *p_i_* represents the model’s predicted probability for pixel *i*, *g_i_* denotes the true label (0 or 1) for pixel *i*, *N* is the total number of pixels in the image, and *ϵ* is a small smoothing term used to prevent division by zero. This loss function aims to maximize the overlap area between the predicted mask and the ground truth mask, thereby improving segmentation accuracy.

Furthermore, to better guide the model’s learning at the pixel level, we incorporated Binary Cross-Entropy (BCE) loss. Binary Cross-Entropy loss measures the classification error for each pixel, and its formula is:

(15) $$L_{BCE}=-\frac{1}{N}\sum_{i=1}^{N}\left[g_{i}\log(p_{i})+(1-g_{i})\log(1-p_{i})\right].$$

Finally, we fused the Dice Loss and *BCE* Loss to form the Hybrid Dice-BCE Loss:

(16) $$L_{HDB}=L_{BCD}+L_{Dice}$$

This composite loss function aims to fully leverage the optimization advantage of Dice Loss in handling global overlap for small target regions (such as barcodes), while compensating for its insufficient sensitivity to pixel-level details. Simultaneously, it cleverly combines the ability of *BCE* Loss to provide precise classification guidance at the pixel level, thereby effectively capturing local features. Through this synergistic effect, we can better balance global overlap optimization with local pixel classification accuracy, significantly enhancing the performance and robustness of the RepSAU-Net model in pixel-level barcode region segmentation tasks.

## 3. Results

This chapter provides a detailed overview of the experimental configuration of the proposed model, an analysis of segmentation results across varying data volumes, a performance comparison with mainstream methods, a dedicated Dice Loss analysis, and a computational cost analysis. All experiments are meticulously designed to comprehensively evaluate the performance and efficiency of the RepSAU-Net in the task of barcode image segmentation embedded within wide-field data.

### 3.1. Experimental Configuration

The experimental environment was configured as follows:

All experiments were conducted on a system equipped with two NVIDIA GTX 1080Ti GPUs. The deep learning framework utilized was PyTorch version 0.4.1, with CUDA version 10.0 for GPU acceleration. Synchronous Stochastic Gradient Descent (SGD) was employed as the optimization algorithm. A weight decay of 0.001 and a momentum of 0.9 were set to ensure a stable and efficient training process. The dataset was partitioned into training and validation sets with a ratio of 4:1, respectively. The batch size for model training was set to 8, balancing memory utilization and training efficiency. The learning rate adjustment strategy was initialized at 0.001. It was subsequently reduced to 0.0001 after 50 training epochs and further decreased to 0.00001 at 80 epochs. The training process was terminated at 100 epochs, aiming to achieve optimal performance.

### 3.2. Ablation Experiment

To assess the individual contributions of the self-attention mechanism and the RepVGG re-parameterization strategy to the RepSAU-Net model’s performance, a series of ablation studies were conducted. These experiments aimed to elucidate the impact of each component on the model’s learning efficacy and its generalization capability across varying data scales.

To further evaluate the model’s performance under conditions of both limited and extensive data, RepSAU-Net underwent segmentation tests with different data volumes. This approach helps in understanding the influence of data quantity on the model’s learning outcomes and its ability to generalize to broader and more diverse datasets. While models might be prone to overfitting on small datasets, larger datasets typically demand more robust feature extraction capabilities to handle complex data distributions.

Table 1 (not provided in this text) presents a comparative analysis of barcode segmentation results using 400 samples (drawn from a small dataset) and 30,000 samples (the full dataset). U-Net served as the baseline model, with RepSAU-Net as the comparative model, and the performance was comprehensively analyzed using Dice Loss and Mean Intersection over Union (MIoU) metrics. Dice Loss is a crucial metric for evaluating segmentation accuracy, particularly effective in handling datasets with class imbalance, such as cases where the barcode region occupies a small proportion of the overall image. MIoU quantifies the level of agreement between predicted segmentation and ground truth regions, with higher values indicating improved segmentation accuracy.

As observed from the results (Table 1, as referenced in the original text), the integration of RepVGG and a self-attention mechanism (SAGAN, implied by context) into the U-Net architecture significantly enhanced performance across both small and full datasets, as evidenced by improvements in both Dice Loss and MIoU. On the smaller dataset of 400 samples, RepSAU-Net achieved a test MIoU of 94.01%, marking an improvement over U-Net’s 88.32%. For the full dataset of 30,000 samples, RepSAU-Net demonstrated an even more substantial gain, reaching a test MIoU of 97.87%, compared to U-Net’s 92.43%. This indicates that RepSAU-Net maintains strong performance across different data scales, with its advantages becoming more pronounced as the data volume increases.

The incorporation of RepVGG, which utilizes a re-parameterization technique to combine multi-branch structures during training into a single, efficient convolutional layer for inference, and the self-attention mechanism, which enables the model to weigh the importance of different parts of the input sequence, led to optimized segmentation results compared to the original U-Net. Specifically, with limited data, RepSAU-Net exhibited superior handling of barcode edges, resulting in more complete segmentation. As the data volume increased, the model was able to identify and segment barcode regions with greater precision. To further align with human visual perception requirements, post-processing techniques involving dilation and erosion algorithms were applied, which further improved the clarity and continuity of barcode boundaries. Experimental results confirm that the model retains its ability to recognize and segment barcode regions even under challenging conditions such as barcode deformation, image compression, variations in size, scaling, stretching, and angular changes. This robustness is critically important for accurately locating hidden information within barcode images embedded in complex backgrounds.

**Figure 4:**
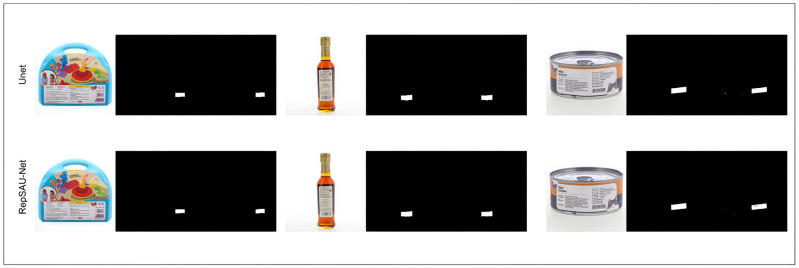
Real barcode image segmentation example.

To validate the model’s applicability and generalization capabilities in real-world scenarios, an additional evaluation was conducted on a small test set comprising 50 real-world images from the SBS dataset. On this challenging subset, RepSAU-Net achieved an MIoU value of 89.74%, significantly outperforming U-Net’s 81.20% MIoU. This performance amply demonstrates its robust effectiveness in practical applications. Visual examples of the corresponding segmentation results are presented in Figure 4.

Table 2 (not provided in this text) presents a comprehensive comparison of Dice Loss, Mean Accuracy (Mean Acc), and Mean Intersection over Union (MIoU) for three distinct models during both training and testing phases. This ablation study was meticulously designed to quantify the specific contributions of the self-attention mechanism and the RepVGG re-parameterization strategy to overall model performance.

U-Net, a widely recognized classical segmentation network, generally performs well across various applications. However, its limitations become apparent in complex tasks requiring global contextual understanding, such as barcode segmentation. For U-Net, the training Dice Loss was recorded as 0.26321 ± 0.00759, while the testing Dice Loss was 0.27394 ± 0.01183. The relatively higher Dice Loss values and larger standard deviations indicate significant performance variability across different test samples and suggest inadequacies in handling global dependencies and intricate features. Its Mean Acc was 93.82%, and MIoU was 97.70%.

The introduction of SAU-Net aimed to address these limitations. SAU-Net demonstrated improved performance, with a training Dice Loss of 0.23890 ± 0.00748 and a testing Dice Loss of 0.26983 ± 0.00893, both superior to U-Net. This improvement suggests that the self-attention mechanism contributes to better model convergence. In terms of performance metrics, SAU-Net achieved an MIoU of 97.82%, slightly surpassing U-Net’s 97.70%, although its Mean Acc experienced a marginal decrease to 93.74%. This enhancement underscores the effectiveness of the self-attention mechanism in capturing long-range spatial relationships and improving segmentation capabilities for complex backgrounds or fine features. While it did not lead to a significant boost in overall pixel accuracy, it notably improved the overlap accuracy of segmented regions.

RepSAU-Net, which integrates both the RepVGG re-parameterization strategy and the self-attention mechanism, exhibited the best performance among all evaluated models. Its training Dice Loss was 0.235902 ± 0.00892, and its testing Dice Loss was 0.27289 ± 0.01136. RepSAU-Net further reduced the training-phase Dice Loss, indicating that the RepVGG structure enhances the model’s feature extraction and representation capabilities, enabling it to leverage the self-attention mechanism more effectively for global information processing. Crucially, RepSAU-Net achieved a significantly improved Mean Acc of 94.96% and an MIoU of 98.36%, outperforming both U-Net and SAU-Net. This demonstrates that the synergistic combination of RepVGG and the self-attention mechanism not only optimizes loss convergence during training but also leads to substantial improvements in final segmentation accuracy and regional overlap. Although its testing Dice Loss showed slight fluctuations compared to SAU-Net, its superior performance on the critical MIoU and Mean Acc metrics confirms RepSAU-Net’s enhanced comprehensive feature extraction and global information processing capabilities, achieving higher precision and better global understanding in complex barcode segmentation tasks.

**Figure 5:**
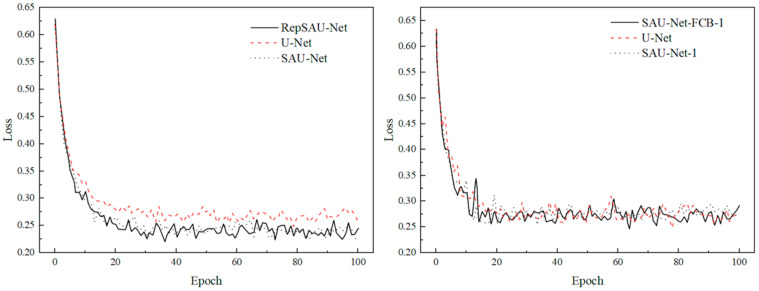
Dice Loss is trained and tested with full data set.

As illustrated in Figure 5 (not provided in this text), RepSAU-Net, by combining RepVGG and the self-attention mechanism, consistently delivered the best performance across all models. Its training Dice Loss of 0.235902 ± 0.00892 and testing Dice Loss of 0.27289 ± 0.01136 represent a further reduction in Dice Loss, particularly during the training phase. This indicates that the RepVGG structure not only augments the model’s feature extraction and representation abilities but also enables it to more effectively utilize the self-attention mechanism for processing global information. While all three models effectively addressed the segmentation task, RepSAU-Net’s superior integrated feature extraction and global information processing capabilities resulted in higher accuracy and a more profound global understanding in the intricate task of barcode segmentation.

### 3.3. Contrast Experiment

**Figure 6:**
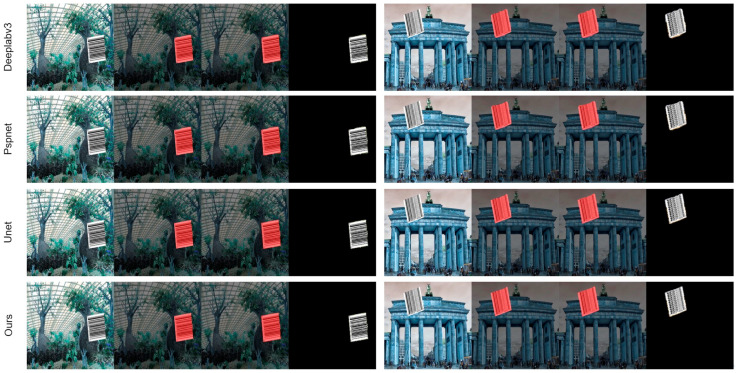
Comparison of segmentation effects of each model (The red segmentation map is for visualization, while the white segmentation map is the segmentation mask).

The comparative experimental results, as presented in Table 3 (not provided in this text), demonstrate the superior performance of the RepSAU-Net network architecture, which is enhanced by the integration of RepVGG and a self-attention mechanism. RepSAU-Net achieved a Mean Intersection over Union (MIoU) of 98.36% and an Accuracy (Acc) of 94.96%. Compared to the FCN-8s model, RepSAU-Net exhibited a remarkable improvement in MIoU by 10.8%. Relative to the PSPNet model, the MIoU increased by 4.93%, while it surpassed the BarcodeNet model by 4.22% and the Deeplabv3 model by 3.45% in MIoU. Even when compared to the original U-Net model, RepSAU-Net showed an MIoU improvement of 0.66% and an accuracy enhancement of 1.14%. These advancements can be attributed to the robust feature extraction capabilities provided by RepVGG and the effective capture of long-range dependencies in images facilitated by the self-attention mechanism. The synergy of these two techniques enables the RepSAU-Net model to achieve greater accuracy and effectiveness in handling image edges and complex textures. Figure 6 (not provided in this text) offers a visual comparison of segmentation outcomes, highlighting that RepSAU-Net produces clearer and more complete segmentation of barcode boundaries compared to other models, thereby demonstrating its exceptional segmentation capability.

Table 4 presents the transfer fine-tuning results of various models on real-world barcode images. RepSAU-Net achieved the highest performance among all models, with a Mean Accuracy (Mean Acc) of 90.17% and an MIoU of 91.25%. This represents an improvement of 1.81 and 1.51 percentage points over SAU-Net for Mean Acc and MIoU, respectively. Compared to other baseline models, RepSAU-Net consistently demonstrated superior performance in terms of MIoU. The most significant improvements were observed over FCN-8s and PSPNet, with MIoU increasing by approximately 9.71 and 6.28 percentage points, respectively, and Mean Acc improving by approximately 9.75 and 4.80 percentage points, respectively. Collectively, these results indicate that RepSAU-Net exhibits significantly superior segmentation accuracy and boundary localization capabilities in real-world barcode scenarios compared to other networks, thereby demonstrating robust transfer generalization performance.

Evaluating model performance also necessitates considering model size (parameter count) and inference speed. Under identical experimental conditions, we conducted an efficiency analysis of RepSAU-Net and other mainstream methods on the SBS dataset, with the results presented in Table 5 (not provided in this text). The FCN-8s model, with a parameter count of 23.73 M, is a relatively lightweight model. In contrast, the PSPNet model has the largest parameter count at 45.92 M, attributable to its complex network architecture. BarcodeNet has a parameter count of 40.69 M, while Deeplabv3 also exhibits a relatively high parameter count. U-Net, with 31.51 M parameters, has slightly more parameters than FCN-8s but fewer than the other models. RepSAU-Net achieves a parameter count of 32.88 M, which is lower than SAU-Net’s 33.72 M. This reduction is due to a core feature of RepVGG—its re-parameterization strategy. During training, RepVGG may incorporate multiple parallel branches, but at the inference stage, these branches are merged into a single convolutional layer through re-parameterization, thereby reducing both parameter count and computational demand. Compared to the baseline U-Net parameter count of 31.51 M, the parameter count of RepSAU-Net shows minimal increase. This is because, while the self-attention mechanism effectively captures global information in images by computing relationships between each position and other positions in the image, it primarily involves multiplication operations of weight matrices without introducing a significant number of additional parameters.

**Figure 7:**
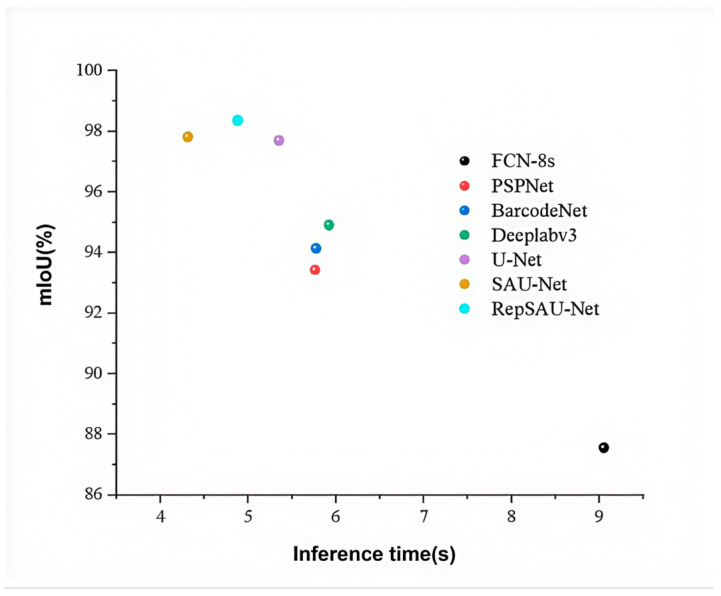
Visualized comparison of prediction time and accuracy on SBS dataset.

In terms of inference speed, the FCN-8s model operates at 9.05 frames per second (inference time), making it one of the faster models in Table 5. PSPNet and BarcodeNet exhibit similar inference time values, reflecting their comparable network complexity and parameter counts. Deeplabv3 achieves an inference time of 5.92, close to that of U-Net. U-Net itself runs at 5.35 inference time, indicating a balance between speed and model size. With the incorporation of the self-attention mechanism, SAU-Net experiences a decrease in inference time to 4.31. RepSAU-Net, however, achieves an inference time of 4.88, an improvement over SAU-Net, demonstrating that even with the addition of the RepVGG structure, its processing speed remains optimized. Compared to the baseline U-Net model, the reduction in inference time for RepSAU-Net is reasonable, considering the increase in model complexity and the corresponding enhancement in segmentation accuracy. Figure 7 (not provided in this text) illustrates a visual comparison of inference time and accuracy across different models on the SBS dataset. The results indicate that the proposed RepSAU-Net model maintains a high inference speed while achieving the highest Mean Intersection over Union (MIoU). This suggests that the model exhibits the highest accuracy in segmenting barcode and non-barcode regions, demonstrating strong recognition and segmentation capabilities for barcode areas.

## 4. Discussion and Conclusions

This study addresses the challenges of insufficient high-quality annotated datasets and suitable deep learning models for semantic segmentation of barcodes in complex backgrounds by proposing a comprehensive solution. Firstly, we synthesized the ScreenBar-Synth (SBS) dataset, which comprises barcodes with hidden information. This dataset was constructed by combining 2000 wide-field Type-A images with 400 Type-B barcode images containing hidden data, resulting in a collection of approximately 30,000 data-embedded barcode images. The proposed synthesis strategy aims to enhance the generalization capability of models, providing a valuable data resource for training deep learning models.

Secondly, we designed and implemented an optimized U-Net-based network architecture named RepSAU-Net, which integrates a self-attention mechanism and the RepVGG re-parameterization strategy. The self-attention mechanism is intended to enhance the model’s ability to capture global information in images, enabling better understanding of the spatial relationships and structural features of barcodes within complex backgrounds. The RepVGG re-parameterization technique aims to improve feature extraction capabilities and inference speed without significantly increasing the parameter count. Experimental results demonstrate that RepSAU-Net performs exceptionally well across various performance metrics. In terms of inference speed, Mean Intersection over Union (MIoU), and Accuracy (Acc), RepSAU-Net achieves high levels of performance, surpassing existing mainstream image segmentation methods. Specifically, RepSAU-Net attains an MIoU of 98.36% and an Acc of 94.96%, effectively enhancing global information capture and feature extraction capabilities during the segmentation of commodity barcode images while maintaining a high inference speed.

The superior performance of RepSAU-Net, reflected in its MIoU of 98.36% and accuracy of 94.96%, is primarily attributed to the synergistic effects of the U-Net architecture, the self-attention mechanism, and the RepVGG re-parameterization strategy. The structure of U-Net, coupled with its skip connections, is inherently well-suited for fusing multi-scale features, which is crucial for segmenting barcodes of varying sizes and resolutions in wide-field images [49,50,51]. The self-attention mechanism further enhances the model’s ability to capture global long-range dependencies, particularly when dealing with small-sized, deformed, or partially occluded barcodes in wide-field scenarios. It effectively correlates disparate barcode pattern segments and distinguishes them from complex backgrounds, overcoming the limitations of traditional CNNs with local receptive fields [52,53,54,55]. Meanwhile, the RepVGG re-parameterization strategy provides a powerful and efficient backbone for feature extraction. By merging multi-branch structures into a single equivalent convolutional layer during inference, RepVGG significantly boosts the model’s representational capacity during training without introducing additional computational overhead during inference. This unique combination ensures that RepSAU-Net not only extracts robust and discriminative features but also effectively integrates global contextual information, achieving high-precision pixel-level segmentation under challenging conditions. Such high precision is critical for subsequent information decoding and recovery, directly addressing the information capacity limitations of traditional barcodes and laying the foundation for broader applications.

The construction of the ScreenBar-Synth (SBS) dataset represents another significant contribution of this study, effectively alleviating the bottleneck of scarce high-quality annotated data in the field of data-embedded barcode segmentation. The automated synthesis strategy proposed in this paper programmatically overlays barcode images with hidden information onto diverse wide-field backgrounds, achieving precise pixel-level automatic annotation. This approach offers a scalable and cost-effective data generation paradigm, significantly outperforming labor-intensive manual annotation. The dataset incorporates carefully designed extensive transformations, including significant scaling, multi-angle rotation, random cropping, and adjustments in illumination and resolution, ensuring that the SBS dataset realistically reflects the complexity and variability of real-world wide-field imaging. Such synthetic diversity is essential for training models with strong generalization capabilities and robustness to real-world environmental factors [56,57,58]. Consequently, RepSAU-Net, trained on the SBS dataset, demonstrates outstanding performance across various conditions, paving the way for practical deployment in industries such as retail and logistics. By enabling reliable segmentation of data-embedded barcodes, this study provides a feasible solution for expanding the data capacity of traditional barcodes without requiring costly upgrades to existing scanning equipment, thereby enhancing commodity information services and optimizing supply chain management.

Despite the significant progress achieved in this study, several avenues for future research warrant further exploration. Although RepSAU-Net achieves an inference speed of 4.88 frames per second (inference time), optimizing the model for faster real-time applications on resource-constrained edge devices remains an important goal. Future work could explore lightweight model architectures, such as leveraging Neural Architecture Search (NAS) or advanced model pruning techniques, to improve efficiency without significantly compromising accuracy [59,60]. Additionally, while the SBS dataset covers a wide range of deformations, extreme real-world challenges—such as severe glare, deep occlusions caused by packaging, or highly textured complex backgrounds—may still pose difficulties for the model [61,62,63]. Future efforts could focus on enhancing the dataset with more adversarial samples or exploring domain adaptation techniques to improve model robustness in such extreme scenarios [64,65]. Another critical direction is the explicit integration of segmentation results with subsequent decoding and information recovery processes. Developing an end-to-end system that not only performs precise segmentation but also robustly decodes hidden information, potentially incorporating feedback mechanisms between segmentation and decoding, would further validate the practical value of this approach. Finally, extending the proposed method to other forms of data-embedded visual encodings or patterns holds the potential to broaden the impact of this research, revolutionizing the traditional barcode domain.

## Figures and Tables

**Table 1:** Comparison of bar code segmentation results with different data volumes.

Data Volume	Method	Train Dice Loss	Train MIoU	Test Dice Loss	Test MIoU
400	U-Net	0.0632	92.06	0.1837	88.32
400	RepSAU-Net	0.0458	95.97	0.1029	94.01
30,000	U-Net	0.0467	97.70	0.1406	92.43
30,000	RepSAU-Net	0.0402	98.36	0.1294	97.87
50 (Real)	U-Net	×	×	0.2313	81.20
50 (Real)	RepSAU-Net	×	×	0.1758	89.74

**Table 2:** Ablation results.

Method	Train Dice Loss	Test Dice Loss	Mean Acc	MIoU
U-Net	0.26321 ± 0.00759	0.27394 ± 0.01183	93.82	97.70
SAU-Net	0.23890 ± 0.00748	0.26983 ± 0.00893	93.74	97.82
RepSAU-Net	0.235902 ± 0.00892	0.27289 ± 0.01136	94.96	98.36

**Table 3:** Evaluation indicators for segmentation of different network structures.

Method	Backbone	Input Size	Mean Acc	MIoU
FCN-8s [45]	VGG-16	480 × 480	87.13	87.56
PSPNet [46]	ResNet-101	473 × 473	92.37	93.43
BarcodeNet	ResNet-101	473 × 473	92.98	94.14
Deeplabv3 [47]	AlignedXception	512 × 512	90.62	94.91
U-Net [48]	U-Net	512 × 512	93.82	97.70
SAU-Net	U-Net	512 × 512	93.74	97.82
RepSAU-Net	U-Net	512 × 512	94.96	98.36

**Table 4:** Evaluation results of transfer fine-tuning of different network structures on real bar code images.

Method	Backbone	Input Size	Mean Acc	MIoU
FCN-8s [45]	VGG-16	480 × 480	80.42	81.54
PSPNet [46]	ResNet-101	473 × 473	85.37	84.97
BarcodeNet	ResNet-101	473 × 473	87.26	86.25
Deeplabv3 [47]	AlignedXception	512 × 512	86.54	86.61
U-Net [48]	U-Net	512 × 512	87.03	88.48
SAU-Net	U-Net	512 × 512	88.36	89.74
RepSAU-Net	U-Net	512 × 512	90.17	91.25

**Table 5:** Analysis of model parameter size and inference time.

Method	Parameters (M)	Inference Time (s)
FCN-8s [45]	23.73	9.05
PSPNet [46]	45.92	5.76
BarcodeNet	40.69	5.77
Deeplabv3 [47]	37.98	5.92
U-Net [48]	31.51	5.35
SAU-Net	33.72	4.31
RepSAU-Net	32.88	4.88

## Data Availability

The original contributions presented in this study are included in the article. Further inquiries can be directed to the corresponding author.
